# Supplementary material for: A phospho-switch controls RNF43-mediated degradation of Wnt receptors to suppress tumorigenesis
Source: Nat Commun. 2020 Sep 15;11:4586. doi: 10.1038/s41467-020-18257-3 (PMC7492264; doi:10.1038/s41467-020-18257-3)
Supplement: Supplementary file 1 — Supplementary Information [file 41467_2020_18257_MOESM1_ESM.docx]

**Inventory of Supplementary Information of Tsukiyama et al.**

**A phospho-switch controls RNF43-mediated degradation of Wnt receptors to suppress tumorigenesis**

Tadasuke Tsukiyama, Juqi Zou, Jihoon Kim, Shohei Ogamino, Yuki Shino, Takamasa Masuda, Alessandra Merenda, Masaki Matsumoto, Yoichiro Fujioka, Tomonori Hirose, Sayuri Terai, Hidehisa Takahashi, Tohru Ishitani, Keiichi I. Nakayama, Yusuke Ohba, Bon-Kyoung Koo and Shigetsugu Hatakeyama

**Supplementary Figures**

Supplementary Figure 1

Supplementary Figure 2

Supplementary Figure 3

Supplementary Figure 4

Supplementary Figure 5

Supplementary Figure 6

Supplementary Figure 7

Supplementary Figure 8

Supplementary Figure 9

Supplementary Figure 10

**Supplementary Methods**

Supplementary Methods provide the information of oligonucleotide sequence used for mutagenesis, qPCR analysis and CRISPR experiments.

**Supplementary Figures**

Supplementary Figure 1 **
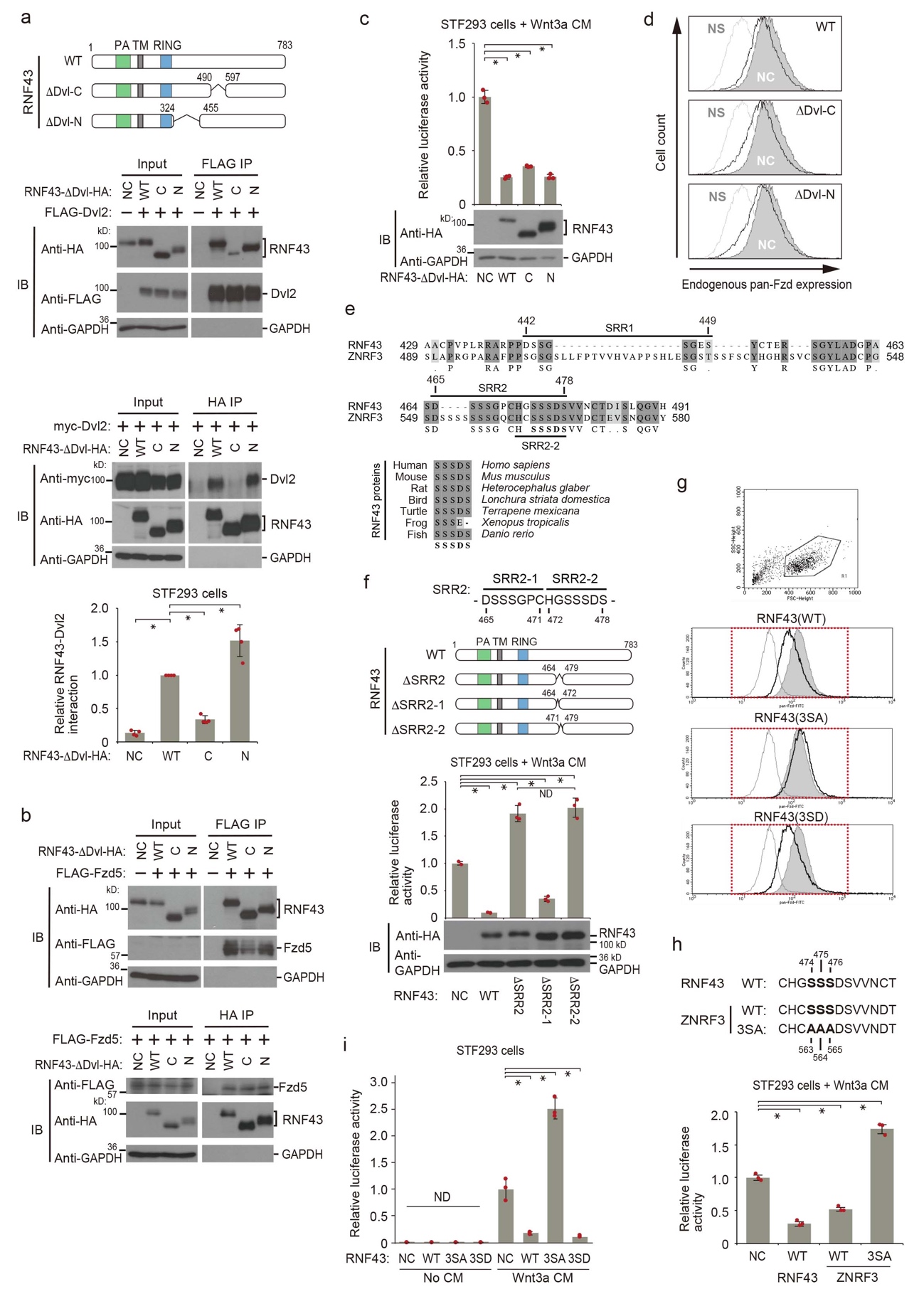
**

**Supplementary Figure 1, Phosphorylation of a conserved serine triplet regulates Fzd-dependent suppression of Wnt/β-catenin signalling independent of Dvl.** **a**, The interaction between RNF43 and Dvl2 was examined in STF293 cells by immunoprecipitation. Deletion mutants used in Supplementary Fig. 1 are illustrated. Relative RNF43-Dvl2 interactions were normalised to Dvl2 expression levels. Binding of RNF43(WT) to Dvl2 was set to 1. Asterisks indicate significant differences from RNF43(WT)-transfected cells. **b**, The interaction between RNF43 and Fzd5 was examined by immunoprecipitation. **c**, Activation of Wnt/β-catenin signalling was examined using STF-luciferase reporter assays following expression of RNF43 mutants. Luciferase activity in empty vector-transfected negative control (NC) cells was set to 1. Expression of RNF43 protein was confirmed by immunoblot analysis. Asterisks indicate significant differences from NC cells. **d**, Surface expression of endogenous Fzd was examined via flow cytometric analysis following expression of RNF43 mutants. Grey lines, NS; not stained (no primary antibodies). Grey filled, NC; blank vector-transfected negative control. Black lines, RNF43 and mutants transfected. FACS data was acquired and displayed with same strategy shown in Supplementary Figure 1g. **e**, The SSSDS sequence of hRNF43(429–491 aa) was aligned with that of hZNRF3(489–580 aa) or of RNF43 sequences from other species. Dark grey boxes denote conserved amino acids. Grey boxes denote similarity. Lines denote the position of SRR-motifs. **f**, Activation of Wnt/β-catenin signalling was examined using STF-luciferase reporter assays following the expression of RNF43 mutants. Deletion mutants used are illustrated. Expression of RNF43 protein was confirmed by immunoblot analysis. Luciferase activity in empty vector-transfected NC cells was set to 1. **g**, Surface expression of endogenous Fzd was examined using flow cytometric analysis following expression of RNF43 phospho-mutant forms. Grey lines, NS. Grey filled, NC. Black lines, RNF43 transfected. Red dashed area indicates the area shown in Fig. 1d. All other FACS data in this study was also acquired and shown in the same way. **h**, Activation of Wnt/β-catenin signalling was examined using STF-luciferase reporter assays following expression of ZNRF3 phospho-mutant forms. The position of ZNRF3 amino acids replaced is as shown. Luciferase activity in empty vector-transfected NC cells was set to 1. Asterisks indicate significant differences from NC cells. **i**, Activation of Wnt/β-catenin signalling was examined using STF-luciferase reporter assays following the expression of RNF43 in the absence or presence of Wnt3a CM. Luciferase activity in empty vector-transfected NC cells with Wnt3a CM was set to 1. Asterisks indicate significant differences from NC cells. Bar graphs and error bars in this figure represent mean ± standard deviation (sd) of at least 3 biologically independent experiments.　Red circles indicate individual values of each sample. The *P* values for the indicated comparisons were determined by one-way ANOVA (*P* < 0.05). n = 3 (c, f, h, i), n = 4 (a) biologically independent samples. Asterisks or ND indicates significant or no significant difference, respectively.

Supplementary Figure 2
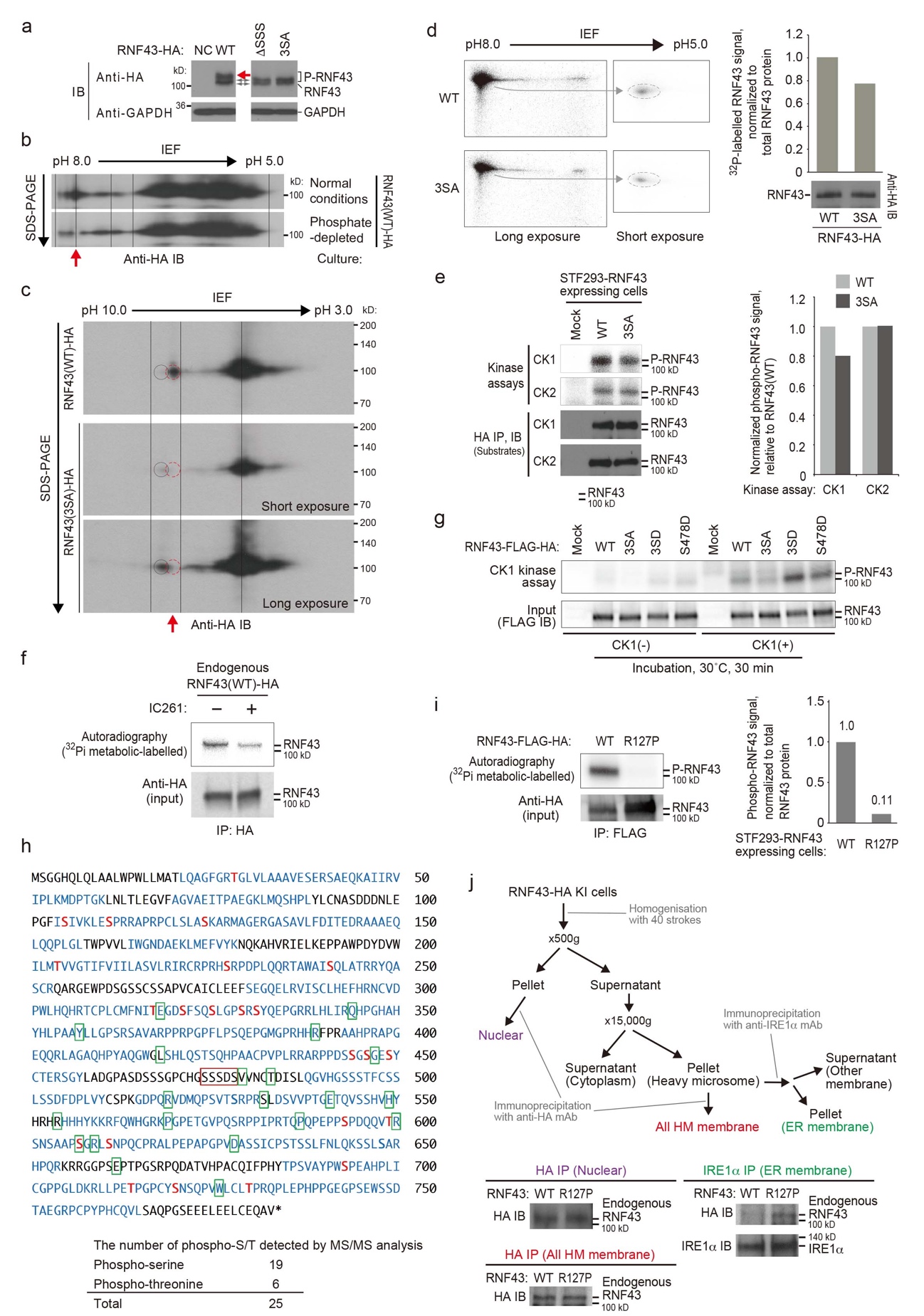


**Supplementary Figure 2, CK1 phosphorylates the serine triplet as part of the multi-step phosphorylation of RNF43.** **a**, Phosphorylation of RNF43 was examined using Phos-tag SDS-PAGE. Red arrow, position of RNF43 phosphorylated specifically in the presence of serine triplet. Black arrows, positions of other phospho- or non-phospho-forms of RNF43. **b**, Phosphorylation of RNF43 was examined by 2D-PAGE and immunoblotting under normal or phosphate-depleted culture conditions. Red arrow, position of phosphorylated RNF43. **c**, Phosphorylation of RNF43 was examined via 2D-PAGE and immunoblotting under normal culture conditions. Red arrow, position of phosphorylated RNF43. Red dashed circles indicate phosphorylated RNF43. **d**, Phosphorylation of RNF43 was examined using ^32^P_i_ metabolic labelling and analysis by two-dimensional electrophoresis (2D-PAGE). Proteins examined were those outlined in Fig. 1e. Radio-labelled RNF43 signals (dashed circles) were normalised to the total RNF43 protein level. The phosphorylation level of RNF43(WT) was set to 1 (n = 1). **e**, Phosphorylation of RNF43 was examined by in vitro kinase assay using ^32^P-γATP and CK1 or CK2. Phosphorylated RNF43 levels were normalised to total RNF43 protein levels. The phosphorylation level of RNF43(WT) was set to 1 (n = 1). **f**, A CK1 inhibitor and ^32^P_i_ metabolic labelling were used to examine the endogenous phosphorylation of RNF43 in HA tag knock-in cells via IP-immunoblotting (see Supplementary Fig. 3). **g**, Phosphorylation of RNF43 and phospho-mutant forms was examined by in vitro kinase assay with ^32^P-γATP in the presence or absence of CK1. **h**, RNF43 phosphorylation in cell lysates was examined via MS/MS analyses. MS/MS experiments were performed as three independent biological replicates. The peptides and the phospho-S/T residues detected are shown in blue and red letters, respectively. The sequence under investigation in this study (SSSDS) or mutations found in tumours and examined further in Supplementary Fig. 6a are denoted by red or green boxes, respectively. **i**, Phosphorylation of the RNF43(R127P) mutant was examined using ^32^P_i_ metabolic labelling. Radio-labelled RNF43 signals were normalised to the total RNF43 protein level. The phosphorylation level of RNF43(WT) was set to 1 (n = 1). **j**, ER localization of endogenous RNF43(R127P) mutant in HA tag knock-in STF293 cells was examined via the fractionation of cellular compartments and IP-immunoblot with anti-IRE1α and anti-HA antibodies.

**
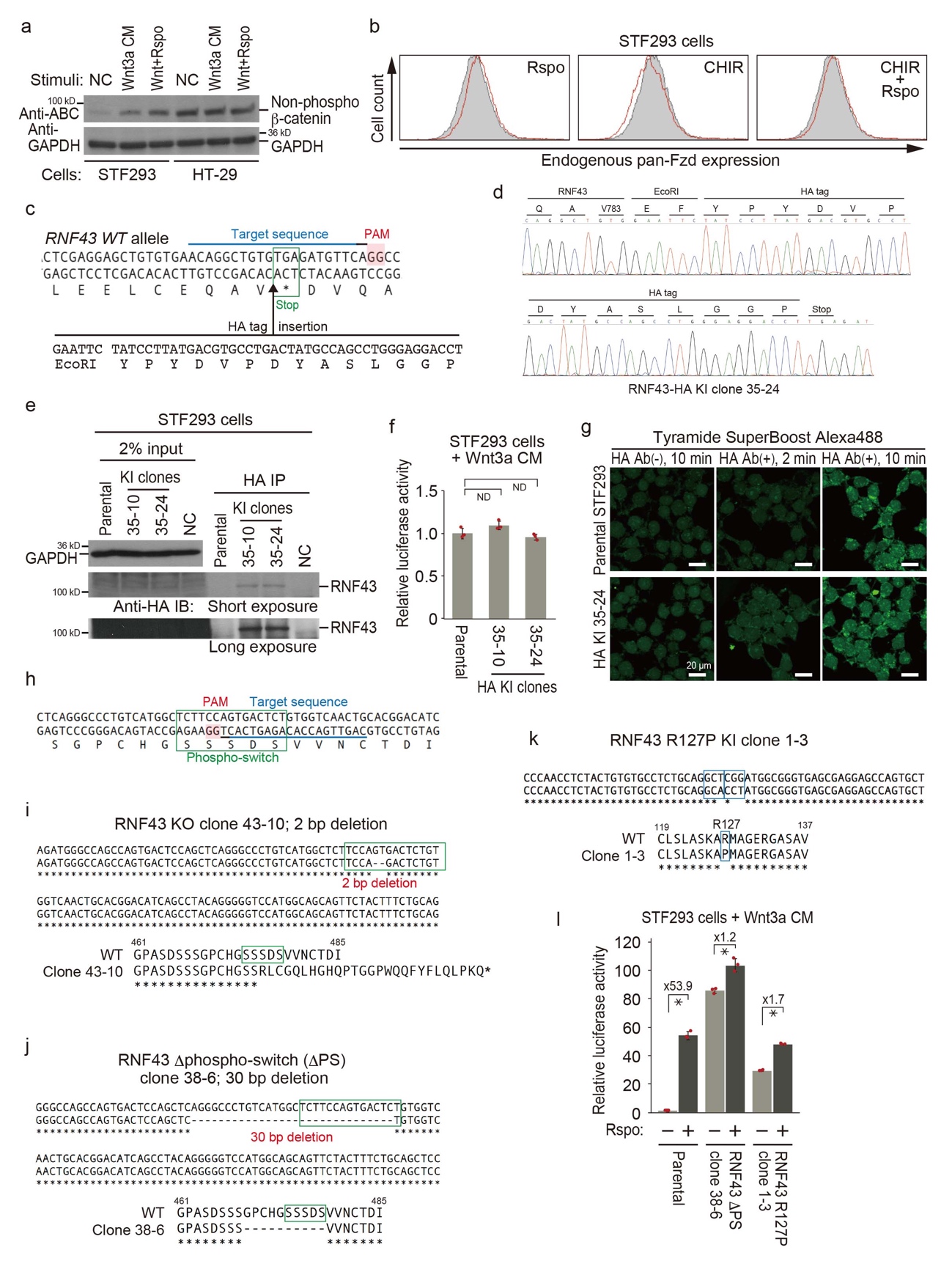
**Supplementary Figure 3

**Supplementary Figure 3, Exogenous RNF43 functions similarly to endogenous RNF43.** **a**, Wnt-Rspo dependent activation of Wnt signalling was examined in STF293 cells and HT-29 cells by immunoblotting with anti-active β-catenin (ABC) Abs at 3 h after stimuli. **b**, Surface function of endogenous RNF43/ZNRF3 was examined in STF293 cells at 24 h after treatment with CHIR and/or Rspo. Surface expression of Fzd protein was detected via FACS analysis with anti-pan-Fzd Abs. Grey filled, NC; DMSO-transfected negative control. Red lines, Rspo and/or CHIR treated cells. FACS data was acquired and displayed with same strategy shown in Supplementary Figure 1g. **c**–**g**, A full-length HA tag (14 amino acids) was inserted genetically just before the stop codon (green box) of RNF43 in STF293 cells using the CRISPR technique. **c**–**d**, Schematic of HA tag insertion (**c**) and genomic sequence of the RNF43-HA knock-in in STF293 cells (**d**). **e**, The expression of endogenous RNF43 protein was confirmed by immunoblotting (IB) and an immunoprecipitation (IP)-IB analysis with anti-HA Abs. The expression of endogenous RNF43 protein remained below the detection limit for a simple IB analysis (left side). The purification and concentration of RNF43 protein from 1.5 x10^7^ cells by IP with anti-HA Abs enabled detection of this protein (right side). f, HA-tagged endogenous RNF43 was subjected to a functional STF-Luc assay. The luciferase activity in parental STF293 cells was set to 1. **g**, The subcellular localisation of endogenous RNF43 was examined via immunofluorescent (IF) staining with an anti-HA mAb and the Tyramide SuperBoost system. However, an endogenous RNF43-HA protein signal was not detected specifically due to a low level of expression. White scale bars, 20 μm. **h**–**k**, RNF43 phospho-switch (PS) mutations and an established oncogenic RNF43 mutation were genetically introduced in STF293 cells using the CRISPR technique. Schematic of the deletion (**h**) and the genomic (upper) and protein (lower) sequences of the RNF43 knockout (KO) (**i**), RNF43 ΔPS (**j**) and R127P mutation (**k**) in STF293 cells. Green box in **h**–**k** or blue box in k indicate the position of PS or R127, respectively. RNF43 KO cells may not express a truncated RNF43 mutant protein due to NMD. **l**, The STF-Luc assay was applied to RNF43(ΔPS) and (R127P) mutant cells with/without Rspo and Wnt3a CM. The luciferase activity in parental STF293 cells without Rspo was set to 1. Asterisks indicate significant differences relative to Rspo(-) cells. Endogenous RNF43 functions on cell surface and (ΔPS) mutant functions as a dominant negative mutant, as shown in Fig. 1 and 2 and Supplementary Fig. 1 and as reported previously via the expression of exogenous non-functional RNF43 mutants. Bar graphs and error bars in this figure represent mean ± standard deviation (sd) of 3 biologically independent experiments. Red circles indicate individual values of each sample. The *P* values for the indicated comparisons were determined by one-way ANOVA (*P* < 0.05). n = 3 biologically independent samples. Asterisks or ND indicates significant or no significant difference, respectively.

Supplementary Figure 4

**
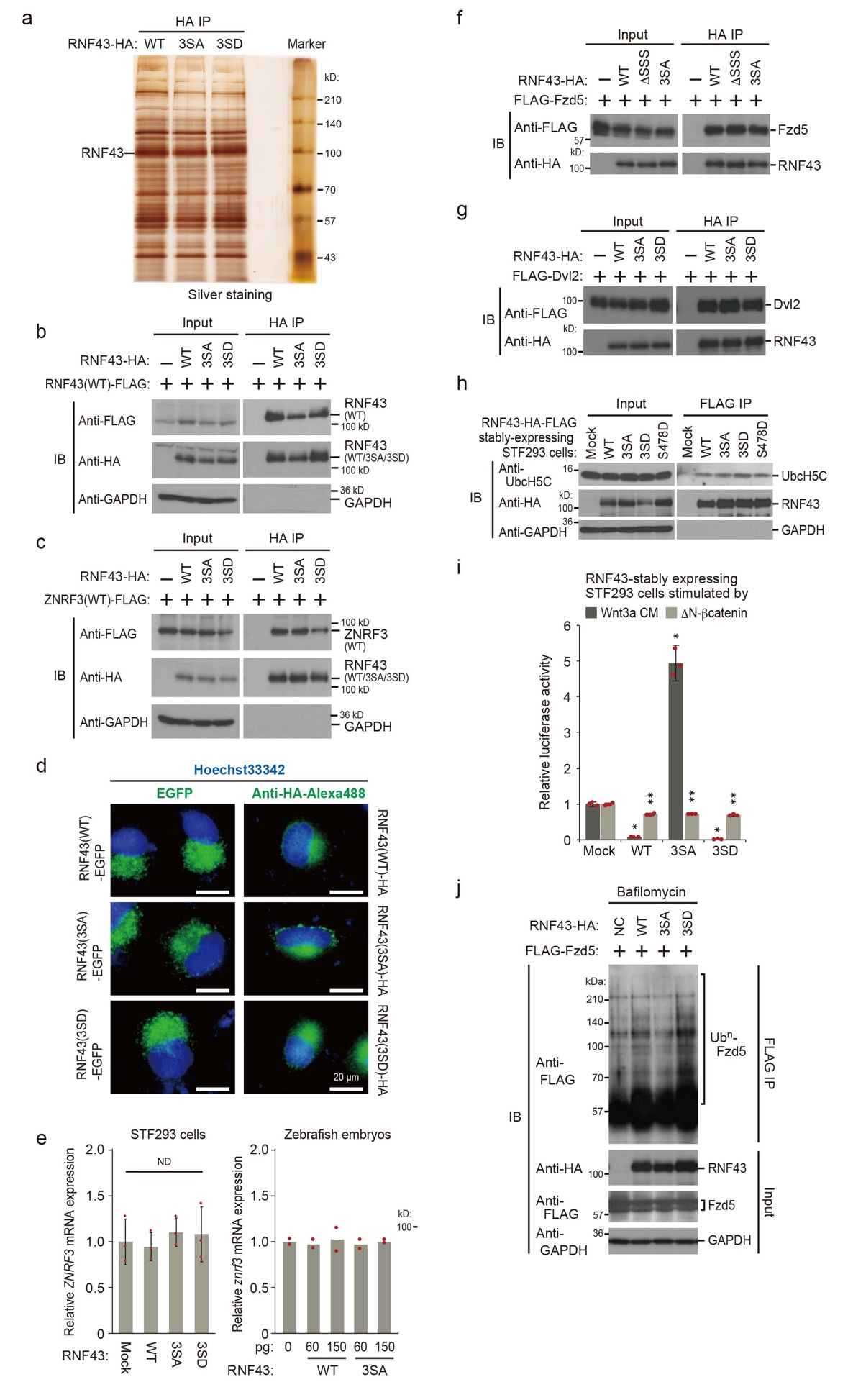
**

**Supplementary Figure 4, Phosphorylation regulates the ubiquitin ligase activity of RNF43.** **a**, Unidentified interactors binding to RNF43 phospho-dependently were investigated using immunoprecipitation and silver staining. **b**, **c**, Formation of homodimer of RNF43 (**b**) and heterodimer of RNF43 with ZNRF3 (**c**) was examined by immunoprecipitation with RNF43 phospho-mutants. **d**, Subcellular localisation of RNF43 mutants was examined with RNF43-EGFP fusion protein or with HA-tagged RNF43 via immunofluorescent staining. Scale bars, 20 µm. **e**, The expression of *ZNRF3/znrf3* mRNA was examined by qPCR in STF293 cells stably expressing RNF43 or in developing Wnt reporter zebrafish embryos at 50% epiboly following the expression of RNF43 mutant forms (n = 25–30). The amount of mRNA for *ZNRF3/znrf3* gene in mock cells (STF293 cells, mean ± sd, n = 3, biologically independent replicates) or in uninjected embryos (zebrafish embryos, mean, n = 2, biologically independent replicates) was set to 1. Red circles indicate individual values of each sample (cells) or pool (zebrafish embryos). All reactions were performed in technical triplicates (both), and results represent biological triplicates (cells) or duplicates (zebrafish embryos). The *P* values for the indicated comparisons in STF293 cells were determined by one-way ANOVA (*P* < 0.05). ND, no significant difference. **f**, **g**, The interaction between RNF43 mutants and known interactors Fzd5 (**f**) or Dvl2 (**g**) was examined by immunoprecipitation. **h**, The interaction between RNF43 mutants and UbcH5C was examined by immunoprecipitation. **i**, The functions of RNF43 phospho-switch mutants were examined using a STF-Luc reporter assay with Wnt 3a CM and ΔN-β-catenin (mean ± sd). Luciferase activity in mock cells was set to 1. Red circles indicate individual values of each sample. Single or double asterisks indicate significant differences from Wnt3a CM or ΔN-β-catenin expression (*P* < 0.05, one-way ANOVA, n = 3 biologically independent replicates). **j**, Ubiquitination of Fzd5 by RNF43 phospho-mutants was examined by IP-immunoblot experiments after lysosome inhibition with bafilomycin A_1_.

Supplementary Figure 5


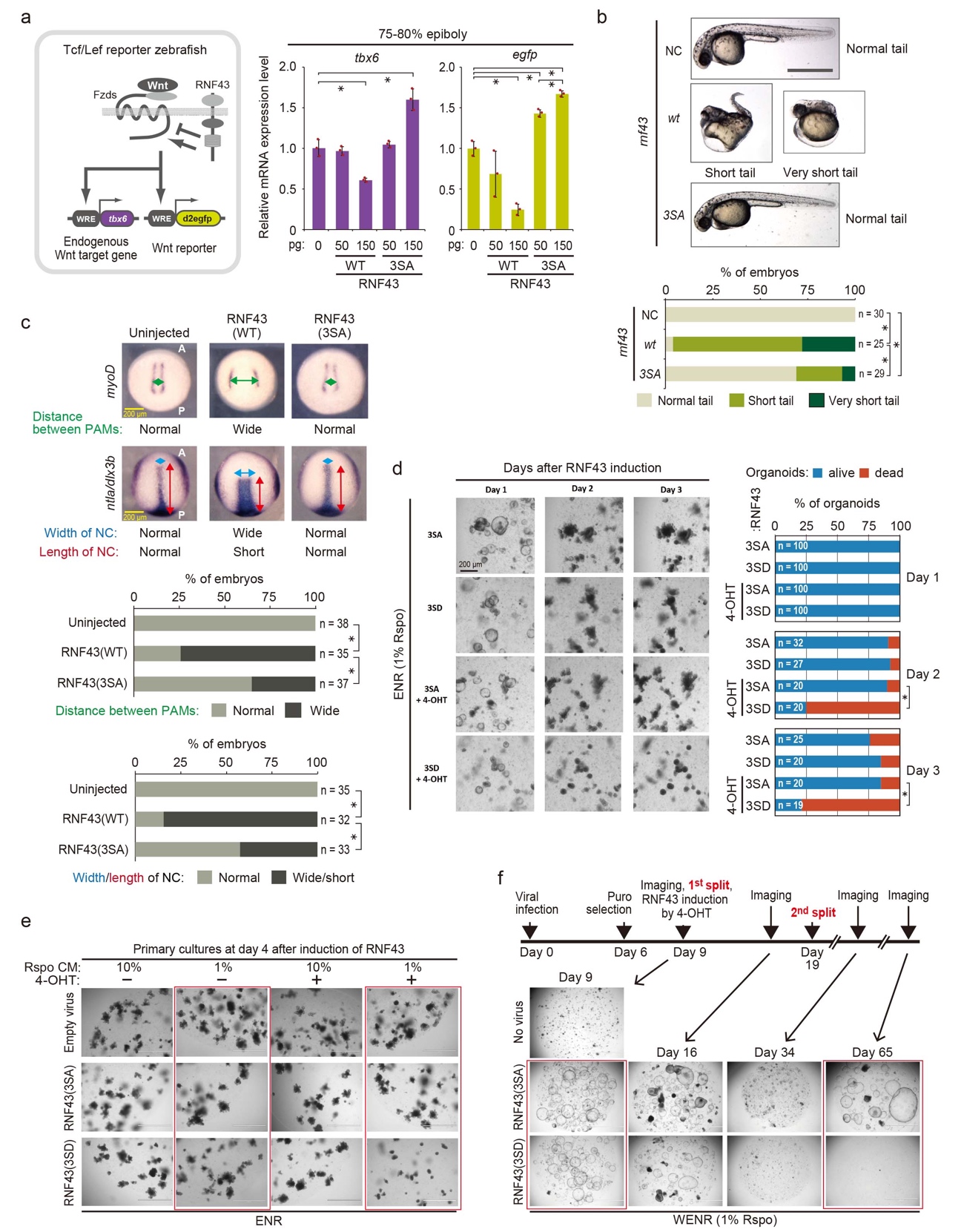


**Supplementary Figure 5, Phospho-regulation of the serine triplet of RNF43 is essential to fine-tune Wnt signalling pathways during zebrafish development and for maintenance of intestinal stem cells.** **a**, Schematic of Wnt reporter zebrafish with RNF43 expression is shown. Expression of the endogenous Wnt target gene (purple), *tbx6* and the reporter gene (green), *egfp* were examined using qPCR in zebrafish embryos 8 hpf following the expression of RNF43 mutant forms. The amount of mRNA for each gene in uninjected embryos was set to 1. Bar graphs and error bars represent mean ± standard deviation (sd) of 3 biologically independent experiments. Red circles indicate individual values of each pools. **b**, Development of zebrafish expressing RNF43 phospho-mutant forms was evaluated at 35 hpf and graphed. **c**, A phenotype termed short and wide, resulting from aberrant non-canonical Wnt signalling, was assessed by *in situ* hybridisation for *myoD* or *ntla* and *dlx3b* following the expression of RNF43(WT) and RNF43(3SA) mutant forms. Representative images demonstrate measurements of paraxial mesoderm (PAM) distance and of the width and length of notochord (NC), denoted by green, blue and red arrows, respectively. The number of embryos displaying each phenotype was quantified. **d**, **e**, Short-term development of intestinal organoids was examined after 4 days following the induction of RNF43 and phospho-mutant forms under ENR conditions containing different Rspo CM (**e**). Red boxed images are shown in Fig. 3c. Organoid growth was evaluated at days 1–3 after transgene induction (**d**). **f**, Experimental timetable is illustrated. Long-term maintenance of intestinal stem cells was examined at day 65 following the induction of RNF43 and phospho-mutant forms under WENR conditions. Red boxed images are shown in Fig. 3d. Asterisks indicate significant differences (*P* < 0.05, one-way ANOVA, n = 3 biological replicates with pools of 22-34 embryos (**a**), with n = 25–30 embryos (**b**, **c**) or with n = 19–100 organoids (**d**)) on indicated comparisons. Scale bars, 1 mm (**b**, **e**, **f**), 200 µm (**c**).

Supplementary Figure 6

**
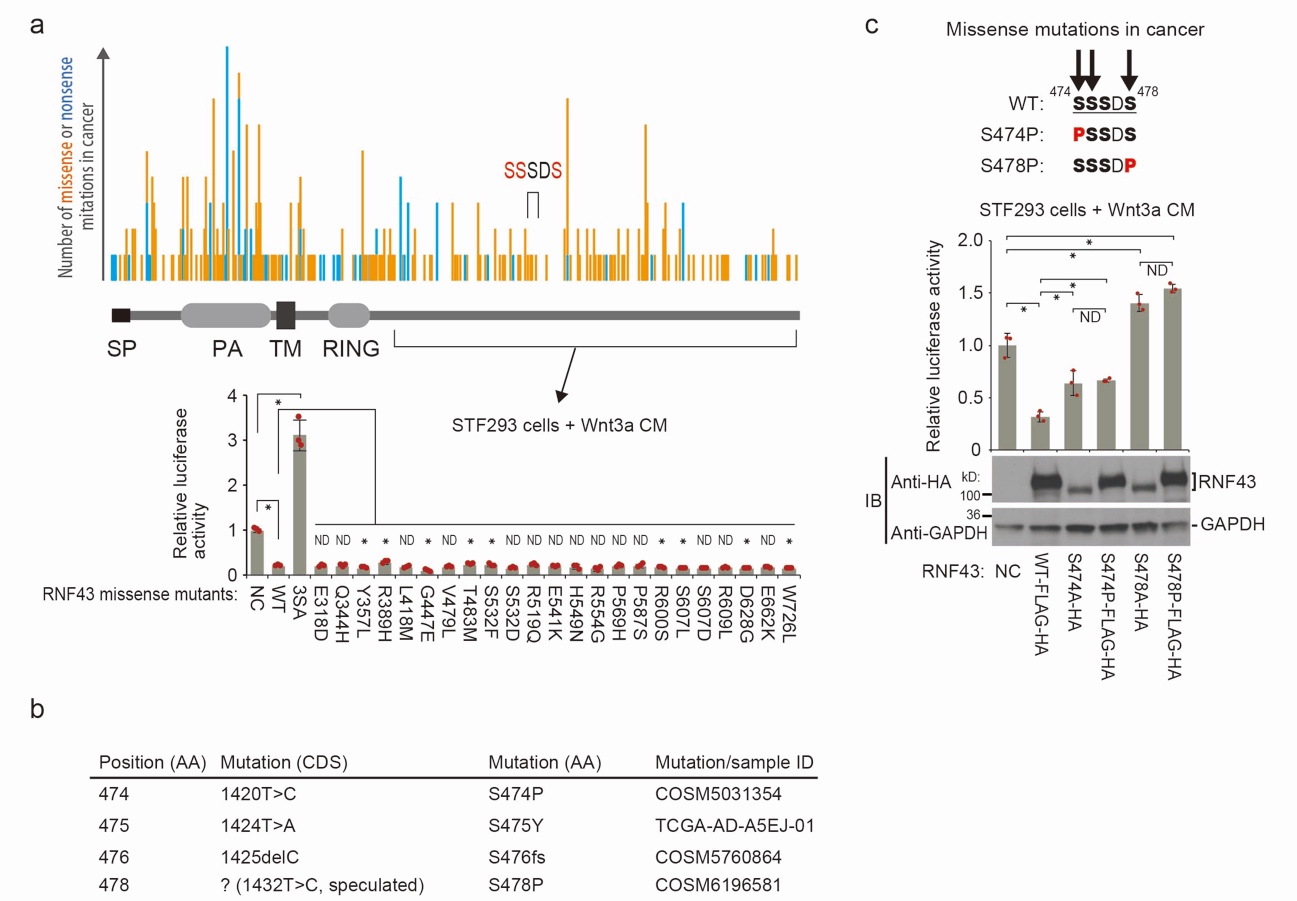
**

**Supplementary Figure 6, Phospho-site missense mutations in RNF43 identified in patient tumours lead to increased Wnt activity.** **a**, **c**, Activation of Wnt/β-catenin signalling with RNF43 missense mutations found in tumours in either the intracellular portion (**a**) or the SSSDS sequence (**c**) was examined using STF-Luc reporter assay. Distribution of mutations in RNF43 protein is illustrated. Luciferase activity in empty vector-transfected (NC) cells was set to 1. Bar graphs and error bars represent mean ± standard deviation (sd) of 3 biologically independent experiments. Red circles indicate individual values of each sample. The *P* values for the indicated comparisons were determined by one-way ANOVA (*P* < 0.05). n = 3 biologically independent samples. Asterisks or ND indicates significant or no significant difference, respectively. **b**, Detail of missense mutations within the SSSDS sequence of RNF43 identified in the cancer genome database. AA, amino acid. CDS, coding sequence.

Supplementary Figure 7 **
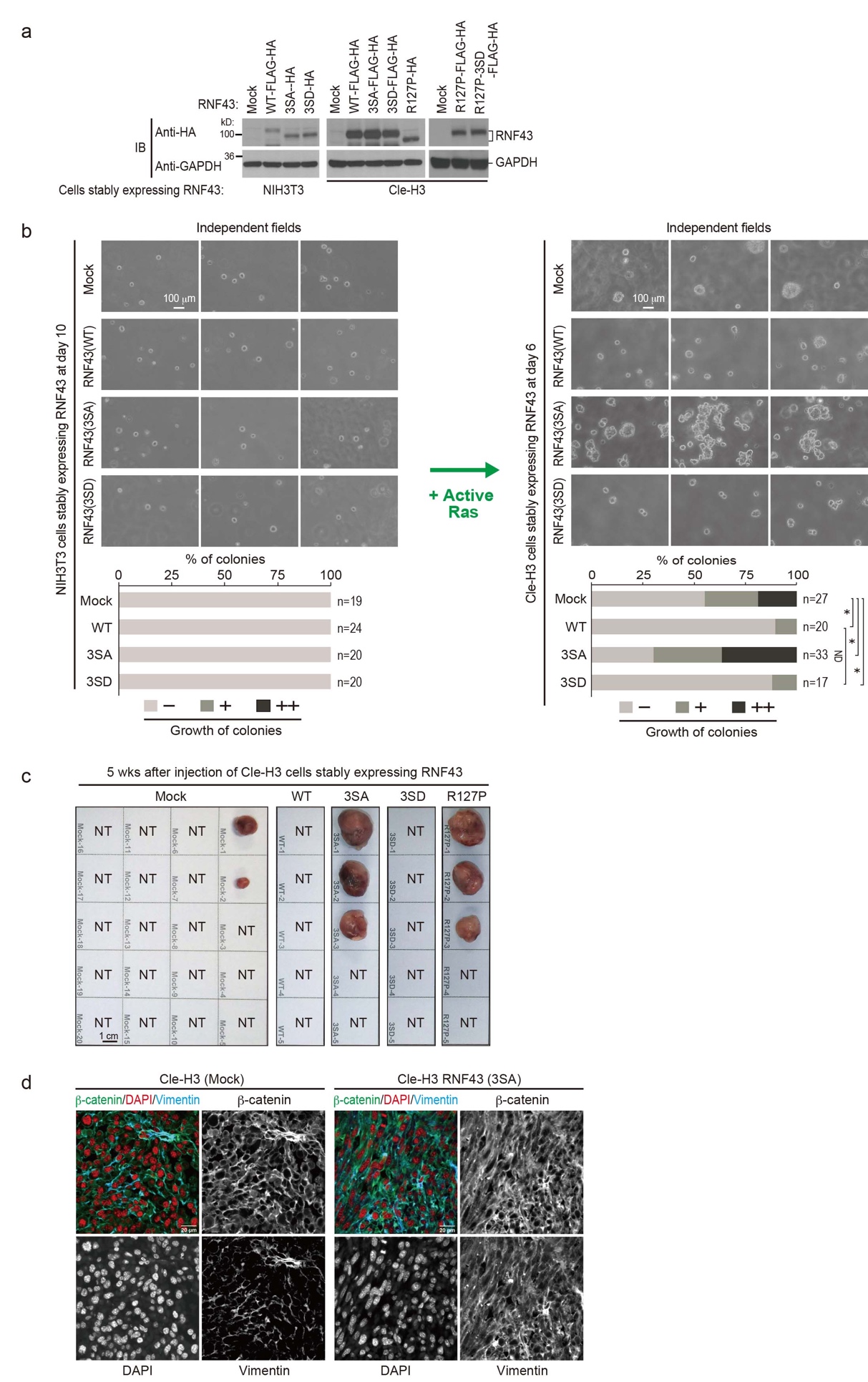
**

**Supplementary Figure 7, Phosphorylation of the serine triplet abrogates tumorigenesis promoted by combined RNF43 mutations with active Ras.** **a**, The expression of ectopic RNF43 proteins in the NIH3T3 and Cle-H3 cell lines used in Fig. 4, 6 and Supplementary Fig. 6 was examined by immunoblotting. **b**, Anchorage-independent colony forming activity was examined via soft agar assay for NIH3T3 or Cle-H3 cells expressing RNF43 phospho-mutant forms. Colony formation was assessed on brightfield microscopy. Asterisks indicate significant differences (*P* < 0.05, one-way ANOVA, n = 29–42) among samples. ND indicates no significant difference. Scale bars, 100 μm. **c**, Tumour growth was examined in nude mice with Cle-H3 cells following the expression of RNF43 phospho-mutant forms at 5 wks after Cle-H3 injection. Images for all the tumours are shown. NT, no tumour observed. **d**, The subcellular localization of β-catenin in tumours illustrated in (**c**) was examined by immunofluorescent staining. Scale bars, 1 cm (**c**), or 20 µm (**d**).

Supplementary Figure 8 **
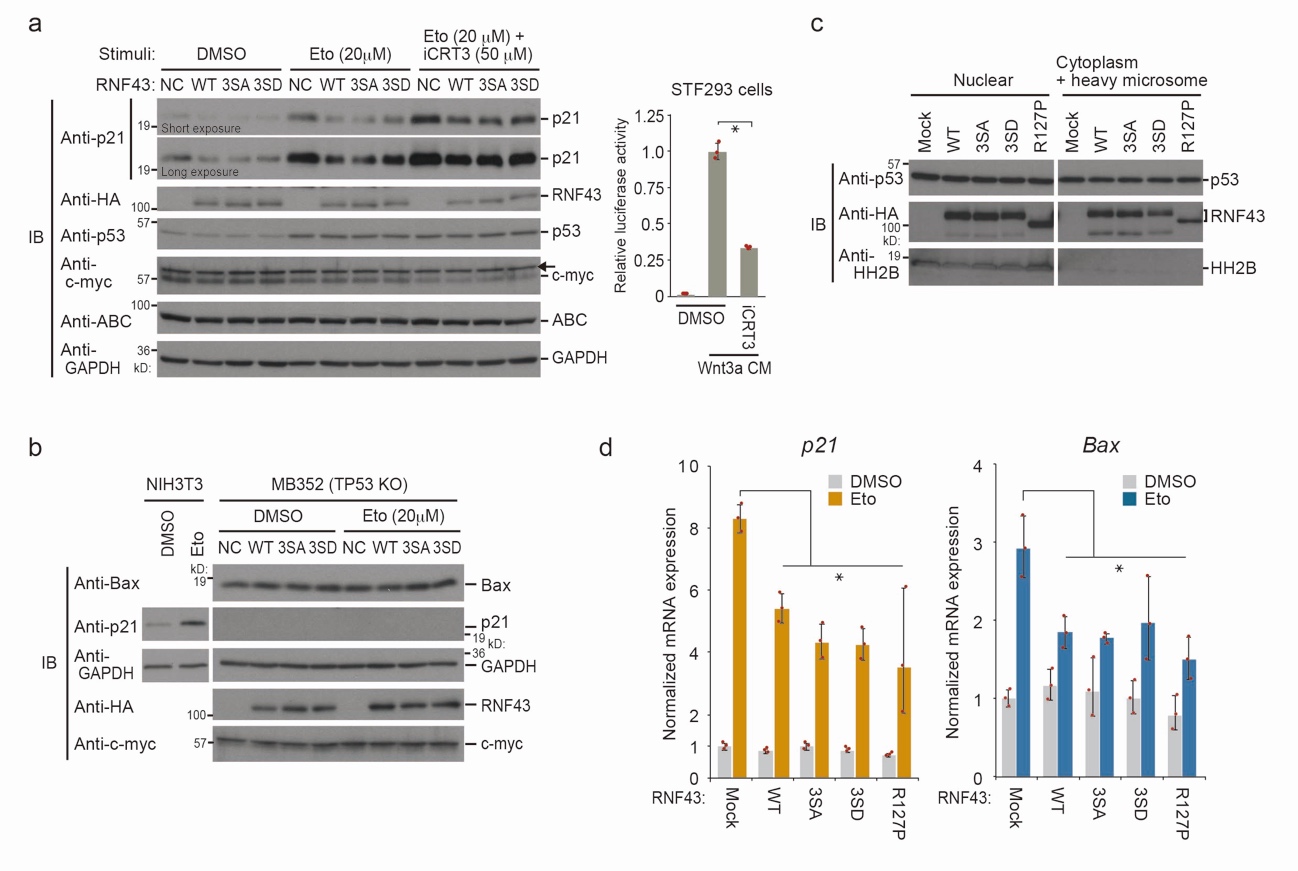
**

**Supplementary Figure 8, Wnt-modulating mutants of RNF43 retain the ability to suppress p53-dependent transcription.** **a**, HCT116 cells expressing RNF43 mutants were cultured with DMSO or etoposide (Eto) in the absence or presence of iCRT3, and expression of p53, non-phosphorylated β-catenin (ABC), c-myc and p21 protein was examined by immunoblotting. Activity of iCRT3 in Wnt suppression was confirmed by STF-luciferase assay. Arrow indicates nonspecific signal. **b**, MB352 cells expressing RNF43 mutants were cultured with DMSO or etoposide (Eto), and expression of c-myc, Bax and p21 proteins was examined by immunoblotting. **c**, Localisation of endogenous p53 protein was examined in nuclear and cytoplasmic/heavy microsome fractions with Eto stimulated HCT116 cells stably expressing RNF43 by immunoblotting. HH2B, histone H2B. **d**, Etoposide-induced expression of p21 and Bax mRNA was examined using qPCR. Expression of p21 and Bax in mock cells with DMSO was set to 1. Bar graphs and error bars in this figure represent mean ± standard deviation (sd) of 3 biologically independent experiments. Red circles indicate individual values of each sample. The *P* values for the indicated comparisons were determined by one-way ANOVA (*P* < 0.05). n = 3 biologically independent samples. Asterisks or ND indicate significant or no significant difference, respectively.

Supplementary Figure 9


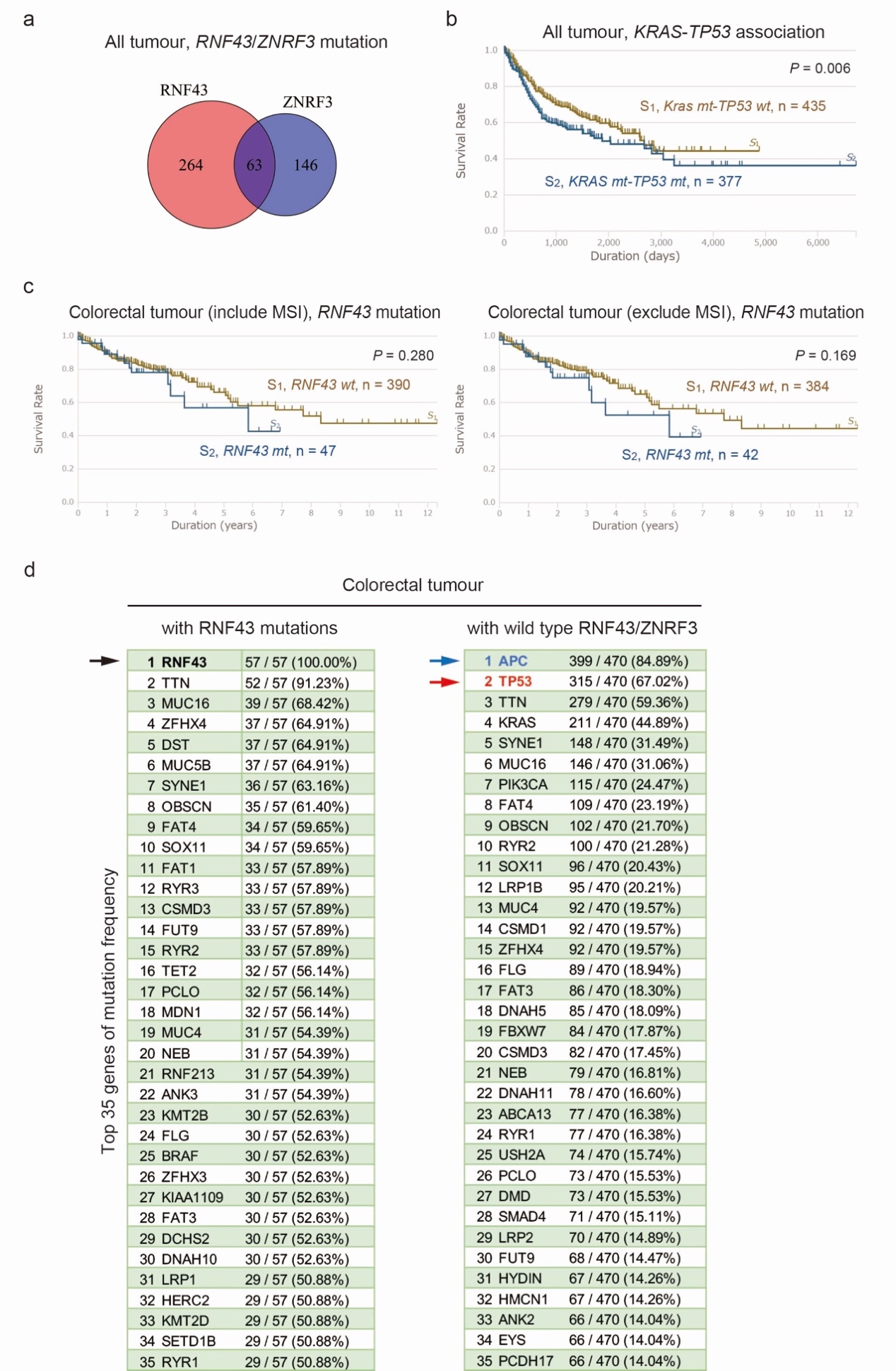
**Supplementary Figure 9, Comprehensive database analysis for the association of genetic alteration in *RNF43*, *KRAS* and *TP53* genes in tumour progression.** **a**, The number of patients who carry a single mutation of *RNF43* or *ZNRF3* and concurrent mutation of these genes in The Cancer Genome Atlas database is shown. **b**, **c**, The survival curve of patients with tumours who carry the mutations indicated was examined via TCGA analysis. Significance between groups of patients with different genetic alteration was calculated using a log-rank test with (left) or without (right) MSI. Number of patients in each group and *P*-value determined from log-rank test are indicated in each graph. Significant difference, *P* < 0.05. **d**, Top 35 genes of additional genetic alterations in tumours that already contain RNF43 mutation or that do not contain mutations in either RNF43 or ZNRF3 are listed. Red, black and blue arrows respectively indicate the position of *TP53, RNF43 and APC* gene in the list. Top 100 genes of each analysis are shown in the Source Data.

Supplementary Figure 10
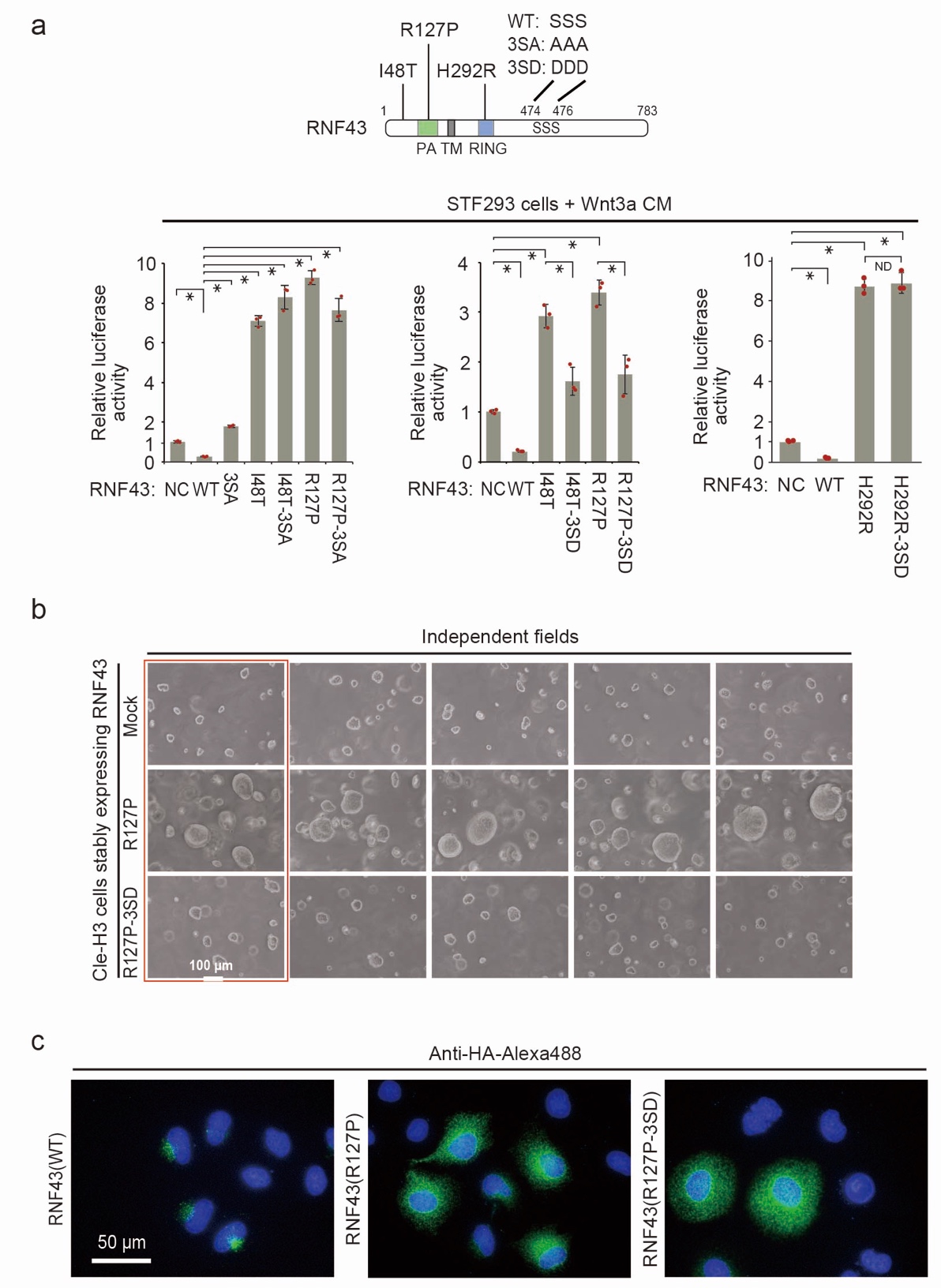


**Supplementary Figure 10, Phosphorylation of conserved serine triplet reverts oncogenic RNF43 phospho-mutant into a tumour suppressor.** **a**, Activation of Wnt/β-catenin signalling with RNF43 mutants was examined using STF-luciferase reporter assays. Schematic of RNF43 mutants used is shown. The luciferase activity in empty vector-transfected (NC) cells was set to 1. Bar graphs and error bars in this figure represent mean ± standard deviation (sd) of 3 biologically independent experiments. Red circles indicate individual values of each sample. The *P* values for the indicated comparisons were determined by one-way ANOVA (*P* < 0.05). n = 3 biologically independent samples. Asterisks or ND indicate significant or no significant difference, respectively. **b**, Anchorage-independent colony forming activity in Cle-H3 cells expressing RNF43 mutants was examined via soft agar assay. Five independent fields are shown. Red box indicates images shown in Fig. 6b. **c**, Subcellular localisation of RNF43 mutants indicated was examined with HA-tagged RNF43 by immunofluorescent staining. Scale bars, 100 μm (**b**), 50 µm (**c**).

**Supplementary Methods**

Supplementary Methods provide the information of oligonucleotide sequence used for mutagenesis, qPCR analysis and CRISPR experiments.

Primers used to generate hRNF43 deletion and point mutants

hRNF43(∆366-441)

5’-GACAGCAGTGGATCTGGAGAAAGC -3’ (forward)

5’-ACCCTCGAGTGCACTCCGGGAAGGGCC-3’ (reverse)

hRNF43(∆366-478)

5’-GTGGTCAACTGCACGGACATCAGC-3’ (forward)

5’-ACCCTCGAGTGCACTCCGGGAAGGGCC-3’ (reverse)

hRNF43(∆SRR1)

5’-TATTGCACAGAACGCAGTGGGTAC-3’ (forward)

5’-AGGGGGCCTGGCCCGGCGTAGGGG-3’ (reverse)

hRNF43(∆SRR2)

5’-GTGGTCAACTGCACGGACATCAGC-3’ (forward)

5’-ACTGGCTGGCCCATCTGCCAGGTA-3’ (reverse)

hRNF43(∆SRR2-1)

5’-CATGGCTCTTCCAGTGACTCTG-3’ (forward)

5’-ACTGGCTGGCCCATCTGCCAGGTA-3’ (reverse)

hRNF43(∆SRR2-2)

5’-GTGGTCAACTGCACGGACATCAGC-3’ (forward)

5’-ACAGGGCCCTGAGCTGGAGTC-3’ (reverse)

hRNF43(∆SSS)

5’-GACTCTGTGGTCAACTGCACGGACATC-3’ (forward)

5’-GCCATGACAGGGCCCTGAGCTGGAGTC-3’ (reverse)

hRNF43(3SA)

5’-GCTGCCGCTGACTCTGTGGTCAACTGC-3’ (forward)

5’-GCCATGACAGGGCCCTGAGCTGGAGTC-3’ (reverse)

hRNF43(S474A)

5’-GCTTCCAGTGACTCTGTGGTCAACTGC-3’ (forward)

5’-GCCATGACAGGGCCCTGAGCTGGAGTC-3’ (reverse)

hRNF43(S474P)

5’-CCTTCCAGTGACTCTGTGGTC-3’ (forward)

5’-GCCATGACAGGGCCCTGAGCTGGAGTC-3’ (reverse)

hRNF43(S475A)

5’- TCTGCCAGTGACTCTGTGGTCAACTGC-3’ (forward)

5’- GCCATGACAGGGCCCTGAGCTGGAGTC-3’ (reverse)

hRNF43(S476A)

5’-TCTTCCGCTGACTCTGTGGTCAACTGC-3’ (forward)

5’-GCCATGACAGGGCCCTGAGCTGGAGTC-3’ (reverse)

hRNF43(3SD)

5’-GATGACGATGACTCTGTGGTCAACTGC-3’ (forward)

5’-GCCATGACAGGGCCCTGAGCTGGAGTC-3’ (reverse)

hRNF43(3SE)

5’-GAAGAGGAAGACTCTGTGGTCAACTGC-3’ (forward)

5’-GCCATGACAGGGCCCTGAGCTGGAGTC-3’ (reverse)

hRNF43(3ST)

5’-ACTACCACTGACTCTGTGGTCAACTGC-3’ (forward)

5’-GCCATGACAGGGCCCTGAGCTGGAGTC-3’ (reverse)

hRNF43(∆Dvl-C)

5’-GGGTACCTGGCAGATGGGCCAGC-3’ (forward)

5’-GACCCCCTGTAGGCTGATGTCCG-3’ (reverse)

hRNF43(∆Dvl-N)

5’-GGGTACCTGGCAGATGGGCCAGC-3’ (forward)

5’-CTGGGAAAATGAATCTCCCTCTG-3’ (reverse)

hRNF43(S478A)

5’-GCTGTGGTCAACTGCACGGACATC-3’ (forward)

5’-GTCACTGGAAGAGCCATGACAGGG-3’ (reverse)

hRNF43(S478D)

5’-GATGTGGTCAACTGCACGGACATC-3’ (forward)

5’-GTCACTGGAAGAGCCATGACAGGG-3’ (reverse)

hRNF43(S478E)

5’-GAGGTGGTCAACTGCACGGACATC-3’ (forward)

5’-GTCACTGGAAGAGCCATGACAGGG-3’ (reverse)

hRNF43(S478P)

5’-CCTGTGGTCAACTGCACGGAC-3’ (forward)

5’-GTCACTGGAAGAGCCATGACAGGG-3’ (reverse)

hRNF43(3SA-S478D)

5’-GCTGCCGCTGACGATGTGGTCAACTGCACGGACATCAGC-3’ (forward)

5’-GCCATGACAGGGCCCTGAGCTGGAGTC-3’ (reverse)

hRNF43(3SD-S478A)

5’-GATGACGATGACGCTGTGGTCAACTGCACGGACATCAGC-3’ (forward)

5’-GCCATGACAGGGCCCTGAGCTGGAGTC-3’ (reverse)

hRNF43(S593A)

5’-GCTCCTGATCAGCAAGTCACCAGATC-3’ (forward)

5’-AGGTGGCTCTGGCTGGGGCTGTGTC-3’ (reverse)

hRNF43(E318D)

5’-GATGGAGATTCATTTTCCCAG-3’ (forward)

5’-TGTGATGTTGAACATGCAGAG-3’ (reverse)

hRNF43(Q344H)

5’-CATCATCCCGGCCATGCCCACTAC-3’ (forward)

5’-GCGAATGAGGTGGAGTCTTCG-3’ (reverse)

hRNF43(Y357C)

5’-TGCCTGTTGGGCCCTTCCCGGAG-3’ (forward)

5’-GGCAGCAGGGAGGTGGTAGTGGGC-3’ (reverse)

hRNF43(R389H)

5’-CACTTCCCCAGAGCTGCACATC-3’ (forward)

5’-GTGATGCCGAGGGCCCATGCCTG-3’ (reverse)

hRNF43(L418M)

5’-ATGAGCCACCTCCAATCCACCTC-3’ (forward)

5’-TCCCCAGCCTTGTGCATAGGGG-3’ (reverse)

hRNF43(G447E)

5’-GAAGAAAGCTATTGCACAGAACGC-3’ (forward)

5’-AGATCCACTGCTGTCAGGGGGC-3’ (reverse)

hRNF43(V479L)

5’-TTGGTCAACTGCACGGACATCAG-3’ (forward)

5’-AGAGTCACTGGAAGAGCCATGAC-3’ (reverse)

hRNF43(T483M)

5’-ATGGACATCAGCCTACAGGGGGTCC-3’ (forward)

5’-GCAGTTGACCACAGAGTCACTGG-3’ (reverse)

hRNF43(S532F)

5’-TTCTTGGACTCGGTGGTGCCCAC-3’ (forward)

5’-ACGAGGCCGAGAGGTCACACTAG-3’ (reverse)

hRNF43(S532D)

5’-GACTTGGACTCGGTGGTGCCCAC-3’ (forward)

5’-ACGAGGCCGAGAGGTCACACTAG-3’ (reverse)

hRNF43(R519Q)

5’-CAAGTGGACATGCAGCCTAGTG-3’ (forward)

5’-CTGGGGATCCCCTTTAGGGCTGC-3’ (reverse)

hRNF43(E541K)

5’-AAAACCCAGGTTTCCAGCCATG-3’ (forward)

5’-CCCTGTGGGCACCACCGAGT-3’ (reverse)

hRNF43(H549N)

5’-AACTACCACCGCCACCGGCACCAC-3’ (forward)

5’-GACATGGCTGGAAACCTGGGTTT-3’ (reverse)

hRNF43(R554G)

5’-GGGCACCACCACTACAAAAAGC-3’ (forward)

5’-GTGGCGGTGGTAGTGGACATG-3’ (reverse)

hRNF43(P569H)

5’-CATGGCCCAGAAACCGGAGTCC-3’ (forward)

5’-CTTCCTGCCATGCCACTGGAAC-3’ (reverse)

hRNF43(P587S)

5’-TCCCAGCCAGAGCCACCTTCTCC-3’ (forward)

5’-CTGTGTCCGAGGAATAGGAGGC-3’ (reverse)

hRNF43(R600S)

5’-AGCTCCAACTCAGCAGCCCCTTC-3’ (forward)

5’-GGTGACTTGCTGATCAGGAGAAG-3’ (reverse)

hRNF43(S607L)

5’-TTGGGGCGGCTCTCTAACCCACAG-3’ (forward)

5’-AGGGGCTGCTGAGTTGGATCTGGTG-3’ (reverse)

hRNF43(D628G)

5’-GGCGCCTCCAGCATCTGCCCCAG-3’ (forward)

5’-AACTGGGCCAGGGGCTGGCTCAG-3’ (reverse)

hRNF43(E662K)

5’-AAGCCCACCCCTGGCTCTCGGC-3’ (forward)

5’-GGAGGGACCCCCCCGCCTTTTC-3’ (reverse)

hRNF43(W726L)

5’-TTGTTGTGCCTGACTCCTCGCC-3’ (forward)

5’-CACTGGCTGTGAATTTGAGTAAC-3’ (reverse)

hRNF43(I48T)

5’-CCAGAGTGATCCCCTTGAAAATG-3’ (forward)

5’-TAATAGCTTTCTGTTCTGCTG-3’ (reverse)

hRNF43(R127P)

5’-CGATGGCGGGTGAGCGAGGAGCC-3’ (forward)

5’-GAGCCTTGCTAGCCAGTGACAG-3’ (reverse)

hRNF43(H292R)

5’-CGTGAGTTCCATCGTAACTGTG-3’ (forward)

5’-GAGGCAGGAAATGACCCGTAGC-3’ (reverse)

Primers used to generate hZNRF3 point mutant

hZNRF3(3SA)

5’-GCCGCTGCTGACTCTGTGGTAGACTGCACTGAGG-3’ (forward)

5’-ACAGTGGCACTGGCCGGAGCTGC-3’ (reverse)

Primers used for qPCR

*egfp*

5’-AGGAGCGCACCATCTTCTT-3’ (forward)

5’-GATGTTGTGGCGGATCTTG-3’ (reverse)

*d-tbx6*

5’-TCCATCCAGACTCACCCGCC-3’ (forward)

5’-AGTGAAGAACCACCAGGCCGT-3’ (reverse)

*d-axin2*

5’-CTTAAACCTGCCACTAAGACCT-3’ (forward)

5’-CATTCTCCTCCATAGCCGTC-3’ (reverse)

*d-nkd1*

5’-CCCAATCCCAAGCATAAG-3’ (forward)

5’-CTCTCCAGGTTCTCATCC-3’ (reverse)

*d-sef1*

5’-TGAGCTCACAGCCCTTCTCA-3’ (forward)

5’-GCAGAAAAGATGGCGGAAAG-3’ (reverse)

*d-id1*

5’-AATGCAAGATCCCGCTGCTG-3’ (forward)

5’-CTTGTTGGTCGGTAGCGTGG-3’ (reverse)

*d-znrf3*

5’-GTACATCGACGGTGAGGAGTTG-3’ (forward)

5’-AAACAGCACCTGGGTTTCCC-3’ (reverse)

*d-β-actin*

5’-TGGACTTTGAGCAGGAGATGGGAA-3’ (forward)

5’-AAGGTGGTCTCATGGATACCGCAA-3’ (reverse)

*hZNRF3*

5’-GGACCCGAAACCATGCCTC-3’ (forward)

5’-TCTGCACCCTTCACATACACC-3’ (reverse)

*hGAPDH*

5’-CTGGGCTACACTGAGCACC-3’ (forward)

5’-AAGTGGTCGTTGAGGGCAATG-3’ (reverse)

*hBax*

5’-TTTGCTTCAGGGTTTCATCC-3’ (forward)

5’-CAGTTGAAGTTGCCGTCAGA-3’ (reverse)

*hp21*

5’-TGTCCGTCAGAACCCATGC-3’ (forward)

5’-AAAGTCGAAGTTCCATCGCTC-3’ (reverse)

Oligonucleotide sequences for genome-editing

*hRNF43* knockout (KO)

5’-CAGTTGACCACAGAGTCAC-3’ (target sequence)

*hRNF43* R127P knock-in (KI)

5’-GCCTCTGCAGGCTCGGATGG-3’ (target sequence)

5’-GCCCAACCTCTACTGTGTGCCTCTGCAGGCACCTATGGCGGGTGAGCGAGGAGCCAGTGCTGTC-3’ (template ssDNA sequence)

*hRNF43* HA knock-in (KI)

5’-ACAGGCTGTGTGAGATGTTC-3’ (target sequence)

5’-CAATGACCTCTTTCCTCCCCGCTCTCTAAATACAGGCTCAGAGGAGGAACTCGAGGAGCTGTGTGAACAGGCTGTGGAATTCTATCCTTATGACGTGCCTGACTATGCCAGCCTGGGAGGACCTTGAGATGTTCAGGCCTAGCTCCAACCAAGAGTGTGCTCCAGATGTGTTTGGGCCCTACCTGGCACAGAGTCCTGCT-3’ (template ssDNA sequence)
