# Supplementary material for: A phospho-switch controls RNF43-mediated degradation of Wnt receptors to suppress tumorigenesis
Source: Nat Commun. 2020 Sep 15;11:4586. doi: 10.1038/s41467-020-18257-3 (PMC7492264; doi:10.1038/s41467-020-18257-3)
Supplement: Supplementary file 3 — Reporting Summary [file 41467_2020_18257_MOESM3_ESM.pdf]

nature research

Corresponding author(s): Tadasuke Tsukiyama

Last updated by author(s): Jul 31, 2020

Reporting Summary

Nature Research wishes to improve the reproducibility of the work that we publish. This form provides structure for consistency and transparency in reporting. For further information on Nature Research policies, see Authors & Referees and the Editorial Policy Checklist.

Statistics

For all statistical analyses, confirm that the following items are present in the figure legend, table legend, main text, or Methods section.

n/a	Confirmed	
☐	☒	The exact sample size (*n*) for each experimental group/condition, given as a discrete number and unit of measurement
☐	☒	A statement on whether measurements were taken from distinct samples or whether the same sample was measured repeatedly
☐	☒	The statistical test(s) used AND whether they are one- or two-sided<br>*Only common tests should be described solely by name; describe more complex techniques in the Methods section.*
☒	☐	A description of all covariates tested
☐	☒	A description of any assumptions or corrections, such as tests of normality and adjustment for multiple comparisons
☐	☒	A full description of the statistical parameters including central tendency (e.g. means) or other basic estimates (e.g. regression coefficient) AND variation (e.g. standard deviation) or associated estimates of uncertainty (e.g. confidence intervals)
☐	☒	For null hypothesis testing, the test statistic (e.g. *F*, *t*, *r*) with confidence intervals, effect sizes, degrees of freedom and *P* value noted<br>*Give P values as exact values whenever suitable.*
☒	☐	For Bayesian analysis, information on the choice of priors and Markov chain Monte Carlo settings
☒	☐	For hierarchical and complex designs, identification of the appropriate level for tests and full reporting of outcomes
☒	☐	Estimates of effect sizes (e.g. Cohen's *d*, Pearson's *r*), indicating how they were calculated

Our web collection on statistics for biologists contains articles on many of the points above.

Software and code

Policy information about availability of computer code

Data collection	CellQuest Ver. 3.3 (Becton Dickinson) in the FACS analysis, Multi Gauge Ver. 3.0 (Fuji Film) in the autoradiography, FACSDiva Ver. 8.0 (Becton Dickinson) in the cell sorting, DP Controller Ver. 3.1.1.267 (Olympus) and ZEN Black 2011 software (Zeiss) in the immunofluorescence.
Data analysis	CellQuest Ver. 3.3 (Becton Dickinson) in the FACS analysis, Multi Gauge Ver. 3.0 (Fuji Film) in the autoradiography, ImageJ Ver. 1.14o/1.54v in the blotting, DP Manager Ver. 3.1.1.208 (Olympus) in the immunofluorescence, PhotoshopCC/C5 (Adobe) in blotting and immunofluorescence.

For manuscripts utilizing custom algorithms or software that are central to the research but not yet described in published literature, software must be made available to editors/reviewers. We strongly encourage code deposition in a community repository (e.g. GitHub). See the Nature Research guidelines for submitting code & software for further information.

Data

Policy information about availability of data

All manuscripts must include a data availability statement. This statement should provide the following information, where applicable:

- Accession codes, unique identifiers, or web links for publicly available datasets
- A list of figures that have associated raw data
- A description of any restrictions on data availability

Human RNF43, NP_001292473. Human ZNRF3, NP_001193927. Mouse Dvl2, NP_031914. Mouse Fzd5, NP_001036124. Mouse RNF43, NP_766036, Naked-mole rat RNF43, XP_021104324. Bengalese finch RNF43, XP_021396062. Three-toed box turtle RNF43, XP_026514312. Tropical clawed frog RNF43, XP_002935238. Zebrafish RNF43, XP_021332049. Phospho-RNF43 MS/MS analysis, http://proteomecentral.proteomexchange.org/cgi/GetDataset?ID=PXD020598 and http://proteomecentral.proteomexchange.org/cgi/GetDataset?ID=PXD020599.
The datasets generated during and/or analysed during the current study are available from the corresponding author on reasonable request.

Field-specific reporting

Please select the one below that is the best fit for your research. If you are not sure, read the appropriate sections before making your selection.

☒ Life sciences ☐ Behavioural & social sciences ☐ Ecological, evolutionary & environmental sciences

For a reference copy of the document with all sections, see nature.com/documents/nr-reporting-summary-flat.pdf

Life sciences study design

All studies must disclose on these points even when the disclosure is negative.

Sample size	No statistical methods were used to predetermined sample size. Sample size was chosen by following the literature in the field.
Data exclusions	No animals examined were excluded. Dead cells were filtered out electrically in FACS analysis, see flow cytometry reporting summary. We excluded from our analyses unviable embryos. Miss injected embryos were also excluded from analysis.
Replication	Almost of reporter assays, FACS analyses, kinase assays, metabolic labelling experiments were repeated 2-4 times independently. We confirmed that these experiments repeated showed similar results, as detailed in the Statistics and Reproducibility section of Methods.
Randomization	We splitted a dish of cells, a culture of organoids, a group of zebrafish or mice equally and randomly allocated to each of experimental group.
Blinding	All the experiments using zebrafish embryos or intestinal organoids and the subjective evaluation of these data were performed by the persons who were not informed the individual function of RNF43 mutants.<br>Other experiments with objective evaluation, including blotting, FACS, luciferase reporter assay, allograft assay and database analysis were performed by persons who know or do not know the individual function of RNF43 mutants. Because subjective impression of researchers does not affect the results of these objective experiments.

Reporting for specific materials, systems and methods

We require information from authors about some types of materials, experimental systems and methods used in many studies. Here, indicate whether each material, system or method listed is relevant to your study. If you are not sure if a list item applies to your research, read the appropriate section before selecting a response.

Materials & experimental systems

n/a	Involved in the study
☐	☒ Antibodies
☐	☐ Eukaryotic cell lines
☒	☐ Palaeontology
☐	☒ Animals and other organisms
☒	☐ Human research participants
☒	☐ Clinical data

Methods

n/a	Involved in the study
☒	☐ ChIP-seq
☐	☒ Flow cytometry
☒	☐ MRI-based neuroimaging

Antibodies

Antibodies used

Secondary antibodies
Anti-mouse IgG-HRP (1:200000, W4021, Promega)
Anti-Rabbit IgG-HRP (1:200000, W4018, Promega)
Mouse Trueblot Ultra (1:200000, 18-8817-33, Rockland)
Rabbit Trueblot Ultra (1:200000, 18-8816-31, Rockland)
Anti-human IgG-FITC (1:200, 109-095-098, Jackson Immunoresearch)
Anti-mouse IgM-Alexa488 (1:1000, A-21042, Invitrogen)
Anti-mouse IgG-Alexa555 (1:1000, A-21127, Invitrogen)
Primary antibodies
Mouse anti-FLAG mAbs (1:5000, M2, F3165, Sigma)
Mouse anti-FLAG mAbs (1:5000, M5, F4042, Sigma)
Mouse anti-HA mAbs (1:5000, HA.11-16B12, MMS-lOlR, Covance)
Mouse anti-myc mAbs (1:5000, 9E10, PRB-lSOP, Covance)
Mouse anti-GAPDH mAbs (1:10000, 016-25523, 5Al2, Wako)
Mouse anti-Ub mAb (1:1000, sc-8017, P4D1, Santa Cruz Biotechnology)
Human anti-panFzd mAbs (20 µg/ml, OMP-18R5, kindly provided by Dr. Austin Gurney, Oncomed)
Mouse anti-Vimentin mAbs (1:2000, V5255, Sigma)
Mouse anti-beta-catenin mAbs (1:500, 199220, BD-TDL)
Rabbit anti-lREIalpha mAbs (1:1000, 3294, Cell signaling Technology)
Rabbit anti-non-P (active) beta-catenin mAbs (1:1000, 4270, Cell signaling Technology)

Rabbit anti-non-P (active) beta-catenin mAbs (1:1000, 8814, Cell signaling Technology)
Rabbit anti-c-myc pAbs (1:400, sc-764, Santa Cruz Biotechnology)
Mouse anti-p21 mAbs (1:250, sc-6246, Santa Cruz Biotechnology)
Rabbit anti-p21 mAbs (1:1000, sc-64016, Cell signaling Technology)
Rabbit anti-human p21 pAbs (1:1000, 64016, Cell signaling Technology)
Rabbit anti-Bax mAbs (1:250, 5023, Cell signaling Technology)
Rabbit anti-Bax mAbs (1:1000, 14796, Cell signaling Technology)
Mouse anti-p53 mAbs (1:1000, sc-126, Santa Cruz Biotechnology)
Rabbit anti-HH2B (1:500, sc-10808, Santa Cruz Biotechnology)
Mouse anti-HA-Alexa488 (1:1000, A488-101L, Covance)

Validation

All antibodies except anti-panFzd mAb are commercially available and commonly used. These commercially available antibodies were all validated by the producers. Please see the manufacturers' website for the validation information. Anti-panFzd mAb was validated in the previous report (ref. #56).

Eukaryotic cell lines

Policy information about cell lines

Cell line source(s)

NIH3T3, HEK293 HCT-116, HeLa cells were obtained from ATCC. STF293 was obtained from NIH/NCI, USA. Cle-H3 (RCB0549) cells were purchased from RIKEN/BRC Japan. MB352, Plat-E/Plat-A, or Wnt3a/L cells were gifted from Dr. Tsukasa Oikawa, Dr. Toshio Kitamura or Dr. Shinji Takada, respectively. Rspo1/HEK293 cells were established by ourselves.

Authentication

None of the cell lines were authenticated except STF293 cells. STF293 cells were authorized as HEK293 cells by STR validation analysis (performed commercially by Biosynthesis, USA).

Mycoplasma contamination

Absence of mycoplasma contamination in all cells used was confirmed by Vector Gem OneStep mycoplasma detection kit for conventional PCR (11-8025, Minerva Biolabs).

Commonly misidentified lines
(See ICLAC register)

HEK is listed in the ICLAC database. But STF293 derived from HEK293 cells we used in this research were confirmed to be HEK293 by STR validation analysis. STF293 cells is indispensable in this study. Because it is well known that STF293 cells show the complete reactivity for known Wnt stimulation and provide sensitive Wnt-reporter activity, compared to all other common cell lines.

Animals and other organisms

Policy information about studies involving animals; ARRIVE guidelines recommended for reporting animal research

Laboratory animals

Adults zebrafish (Danio rerio) were used to obtain fertilized eggs. Zebrafish embryos and larvae used in this study were at 8 to 10 hours post fertilization and one day post fertilization, respectively.
Vil-CreERT2 mice was provided by Dr. Sylvine Robine and maintained in house. We isolated organoids used in this study from Vil-CreERT2 mice at 8-10 wks of the age. Mice used for the cell line transplantation were 5-6 wks of age female BALB/cAJcl-nu/nu and obtained from CREA Japan. These mice were maintained in SPF under the condition of 22-24°C, 40-60% humidity, 12 h light/12 h dark cycles.

Wild animals

We did not use wild animals.

Field-collected samples

We did not use field-collected samples.

Ethics oversight

Experimental design using mice was approved by NATIONAL UNIVERSITY CORPORATION HOKKAIDO UNIVERSITY PROVISIONS ON ANIMAL EXPERIMENTS (18-0012). Experimental zebrafish care was performed in accordance with institutional (Gunma and Osaka University) and national guidelines and regulations.

Note that full information on the approval of the study protocol must also be provided in the manuscript.

Flow Cytometry

Plots

Confirm that:

☒ The axis labels state the marker and fluorochrome used (e.g. CD4-FITC).

☒ The axis scales are clearly visible. Include numbers along axes only for bottom left plot of group (a 'group' is an analysis of identical markers).

☒ All plots are contour plots with outliers or pseudocolor plots.

☒ A numerical value for number of cells or percentage (with statistics) is provided.

Methodology

Sample preparation

STF293 cells without or with RNF43 expression were subjected to flow cytometric analysis. Those cells were made as single cell suspension with the Cell Dissociation Buffer Enzyme-Free PBS-based (13151, Gibco). Cells were stained with anti-pan-Fzd

	antibodies in 0.05% BSA-PBS (staining buffer, SB) for 45 min on ice, washed x2 times with SB and subsequently incubated with anti-human lgG-FITC for 20 min on ice. Cells stained were subjected to flow cytometric analysis after x2 times of wash.
Instrument	All flow cytometric data were collected using a FACSCalibur (Becton Dickinson).
Software	All of data analyses were performed using a CellQuest Ver. 3.3 (Becton Dickinson) software.
Cell population abundance	More than 10,000 living cells (10,000-20,000) were collected for the evaluation of Fzd expression on cell surface.
Gating strategy	A live cell gate is indicated in Supplementary Fig. lg. We did not show the fluorochrome used, axis scales and the number of cells in each figures independently (Fig. ld, 2d, 2g, Supplementary Fig. 1d, 3b). However, we show those information integratedly inSupplementary Fig. lg, which is applicable to all figures above. Because all FACS data in this study was acquired and displaied using same strategy with in Supplementary Figure 1g.

☒ Tick this box to confirm that a figure exemplifying the gating strategy is provided in the Supplementary Information.
